# Hidden Tunnel: A Case of Duodenal-Colonic Fistula Caused by Cytomegalovirus

**DOI:** 10.7759/cureus.8425

**Published:** 2020-06-03

**Authors:** Rahul Myadam, Yousaf Zafar, Pooja Kalantri

**Affiliations:** 1 Internal Medicine, University of Missouri-Kansas City, Kansas City, USA; 2 Internal Medicine, Naples Community Healthcare, Naples, USA; 3 Internal Medicine, Saint Vincent Hospital, Worcester, USA

**Keywords:** enteric fistula, cytomegalovirus (cmv)

## Abstract

We present the case of an 86-year-old African American gentleman who presented with fatigue, diarrhea, and weight loss. He had elevated liver enzymes in an obstructive pattern. A magnetic resonance cholangiopancreatography scan showed edema around the stomach and duodenum, which prompted evaluation with an esophagogastroduodenoscopy. A large enteric fistula between the duodenum and colon was visible, and biopsies returned positive for cytomegalovirus (CMV). The patient did not have any known risk factors for immunodeficiency and was successfully treated with medical therapy. Our case is unique in the severity of CMV infection in an otherwise healthy individual.

## Introduction

Cytomegalovirus (CMV) is a member of the herpes family of viruses, which generally causes infections in patients with immunosuppression from an organ transplant, acquired immune deficiency syndrome (AIDS), and those receiving chemotherapy. After the advent of highly active antiretroviral therapy, AIDS-related CMV gastrointestinal disease has decreased by sixfold according to one report [1]. Rarely, as in a Chinese study from 1999, CMV can cause end-organ disease (commonly colitis) in patients in whom no apparent cause of immune suppression can be found [2]. An enteric fistula is a rare complication due to CMV, with very few reported cases in the literature so far. Most of the reported cases involve patients with AIDS. Optimal treatment for a CMV enteric fistula is unknown because of the rarity of the condition, especially in patients without AIDS. In this report, we present a patient with CMV duodenal-colonic fistula with no evidence of AIDS and who was treated conservatively with medical management. 

## Case presentation

**Figure 1:**
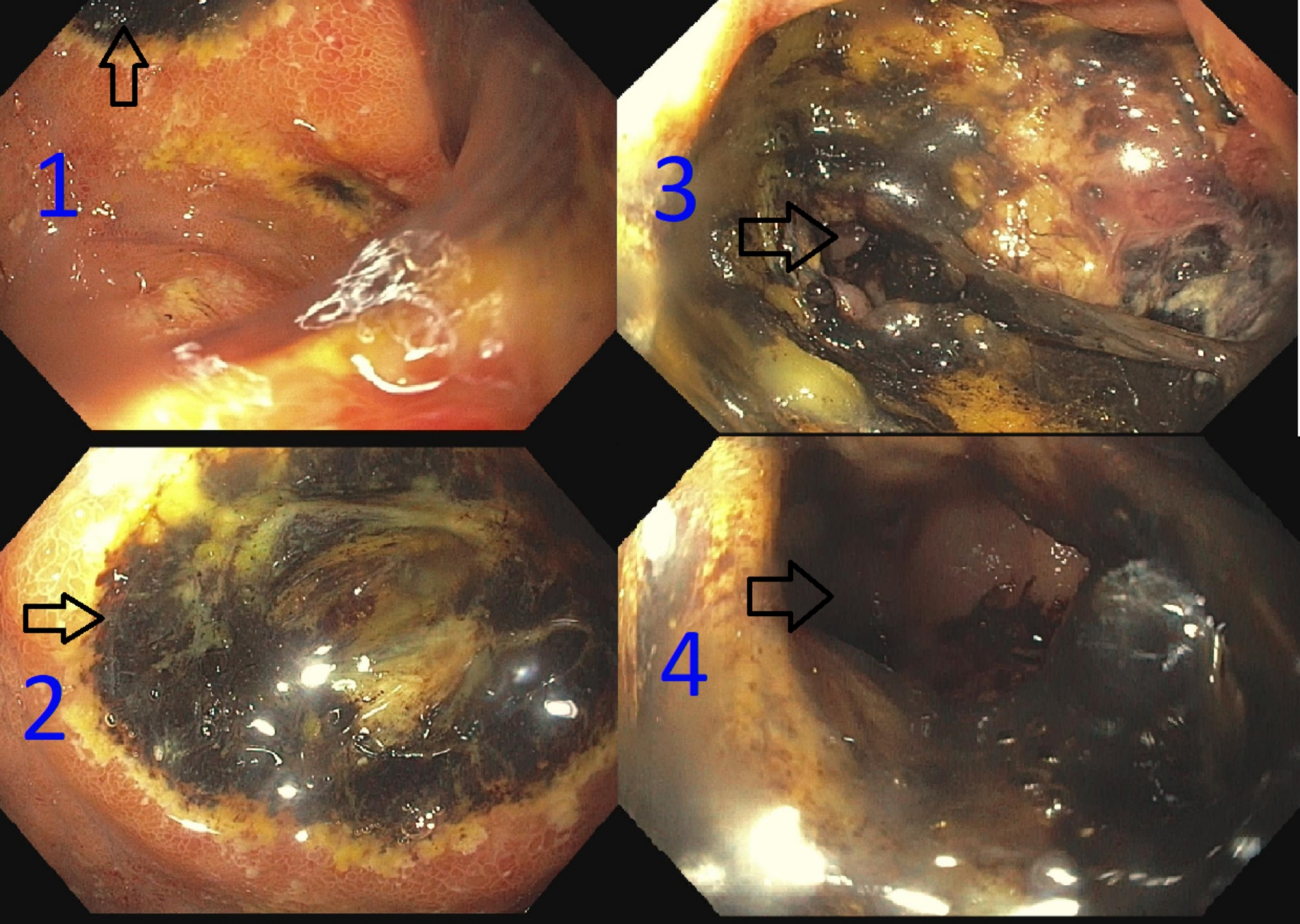
Esophagogastroduodenoscopy Esophagogastroduodenoscopy showing the second part of the duodenum with a necrotic ulcer (1 and 2) and the fistula between the second part of the duodenum and the colon (3 and 4).

A previously healthy 86-year-old African American gentleman presented to the emergency department with complaints of fatigue, jaundice, and non-bloody diarrhea for the previous two months. He also noted significant weight loss in this period. His past medical history was significant for an appendectomy. He denied alcohol use and was not taking any prescription medications. He denied a family history of cancer or liver disease. His initial labs showed elevation of liver enzymes along with mild anemia. His alanine aminotransferase was 93 units/L (normal 7-56 units/L), aspartate aminotransferase was 65 units/L (normal 10-40 units/L), total bilirubin was 3.3 mg/dL (normal 0.2-1.2 mg/dL), and alkaline phosphatase was 200 units/L (normal 33-130 units/L). The viral hepatitis serology was negative. He underwent an ultrasound of the right upper quadrant, which did not show biliary stones. A magnetic resonance cholangiopancretography scan showed edema and hyperemia of the gastric antrum and duodenum, but no obstructive mass or biliary ductal dilation. The gastroenterology team recommended obtaining an esophagogastroduodenoscopy. This procedure showed a large duodenal ulcer with a duodenal-colonic fistula that was large enough for the scope to pass through (Figure 1). There was no evidence of a biliary tumor. The biopsies taken from the duodenum showed ulcerative duodenitis with rare CMV inclusion bodies. No dysplasia or malignancy was found. The Helicobacter pylori biopsy test was negative. The liver biopsy showed bland cholestasis. The blood CMV deoxyribonucleic acid polymerase chain reaction level (PCR) was high (16 copies/PCR; normal 0-5 copies/PCR). The human immunodeficiency (HIV) antibody test was negative. The patient was treated with intravenous ganciclovir, followed by oral valganciclovir for a total of six weeks. A repeat upper gastrointestinal endoscopy was done two months later, and it showed a healing duodenal ulcer but no fistula. The blood CMV level was undetectable at the time of completion of anti-viral treatment.

A few months later, the patient presented to the emergency room with complaints of sudden onset abdominal pain, and was taken urgently to the operating room. He had an internal small bowel hernia with infarcted segments of the ileum and the jejunum. The biopsy of the bowel loops was negative for CMV. A year after the initial presentation, the patient was seen in the immunology clinic to evaluate why he had such a severe infection from CMV despite being apparently immunocompetent. His labs were significant for only mildly reduced B lymphocyte count 47 cells/mm^3^ (normal 100-700 cells/mm^3^), with slightly lower immunoglobulin (Ig) G level 748 mg/dL (normal 762-1,488 mg/dL), and IgM level 25 mg/dL (normal 38-328 mg/dL). The rest of the labs were unremarkable, including a complete blood picture, lymphocyte subsets, and lymphocyte proliferation studies. The CD4 level was 665 cells/mm^3^ (normal 440-2,160 cells/mm^3^). It was felt that the duodenal infection with CMV was an isolated event, and because he had no recurrent infections, it was decided by the patient and the immunologist to forego any further investigations of immune deficiency.

## Discussion

CMV is a ubiquitous pathogen, and approximately 60% to 90% of healthy individuals test seropositive [3]. The CMV infections in healthy adults are either mild or sometimes asymptomatic. However, it is the most common viral opportunistic infection in AIDS and organ transplant recipients [4,5]. In immunosuppressed patients, CMV infection can cause ulcers anywhere in the gastrointestinal tract, but most commonly in the stomach and the colon [6]. However, CMV is rarely associated with gastroduodenal ulcers in immunocompetent patients [6]. Our patient had low B lymphocytes, and his immunoglobulin levels were mildly decreased, but his CD4 level was above the threshold (50 cells/mm^3^) that is usually associated with CMV infection in AIDS patients. He did not have any opportunistic infections before this presentation. A low B-cell immunity could have predisposed him to CMV infection, but this is atypical, as severe CMV infection is usually seen in T-cell-mediated immunodeficiency [7]. In a study done by Ng et al. in patients with CMV colitis and no apparent cause of immunodeficiency, only one patient out of 10 had low B lymphocyte count [2]. The other risk factors associated with CMV gastrointestinal disease in apparently immunocompetent individuals include recent red blood cell transfusion and steroid use, which were not present in our patient [8]. Table 1 lists the various types of CMV enteric fistula described in the literature along with associated risk factors.

**Table 1:** List of CMV enteric fistulae cases reported in the literature and associated risk factors CMV, Cytomegalovirus

Location of CMV enteric fistula	Risk factors for infection
Colo-vesical	AIDS
Entero-colic	AIDS
Cholecysto-duodenal	Oral steroids
Gastro-colic	Chemotherapy
Colo-cutaneous	Kidney transplant
Broncho-esophageal	AIDS
Recto-vaginal	None found
Colo-cutaneous	AIDS

An enteric fistula is an abnormal communication between two epithelial lined surfaces. Depending on the site of communication, a fistula could be entero-enteric, entero-biliary, entero-vesical, entero-vaginal, and entero-cutaneous. The common causes of an enteric fistula include Crohn's disease, tuberculosis, radiation, and previous abdominal surgeries. The CMV gastrointestinal lesions are variable, and range from patchy erythema, exudates, micro-erosions, diffusely edematous mucosa to multiple mucosal erosions, deep ulcers, perforations, and pseudotumor [9]. Only one other case of duodenal ulcer leading to a fistula (cholecysto-duodenal) was previously described in the literature by Mori et al. [10]. The diagnosis of CMV enteric fistula requires both the endoscopic appearance and the characteristic histopathology. The endoscopy aids in the observation of the fistula and in taking the biopsies. Like other sites of CMV infection, histopathological diagnosis requires visualization of giant cells with intranuclear inclusions called Cowdry A bodies (owl’s eye appearance) in the setting of acute inflammatory infiltrate. Other diagnoses that need to be ruled out on biopsy include lymphoma and mycobacterial disease, which were done so in our patient. 

The treatment of CMV fistula is uncertain due to the lack of sufficient data. Anecdotal evidence from isolated case reports noted successful healing of the fistula with medical treatment alone [4,10,11]. The anti-viral treatment consists of an induction and a maintenance phase. The most common regimen reported was ganciclovir 5 mg/kg every 12 hours, followed by oral valganciclovir 900 mg per day for a total of six weeks. In our patient, anti-viral treatment was stopped after six weeks because of negative CMV blood titers. 

The above case was presented as a poster 'Myadam R, Zafar Y, Jamil LH: A Case of Cytomegalovirus Causing Duodeno-Colonic Fistula in Immune Competent Patient' at the American College of Gastroenterology 2019 Annual Scientific Meeting on 10/27/2019, held in San Antonio, Texas. 

## Conclusions

The gastrointestinal disease due to CMV is increasingly being identified in patients without AIDS. An enteric fistula could be rare sequelae of CMV infection that needs to be recognized during endoscopy. An immunology evaluation may be helpful in a patient without any apparent cause of immunodeficiency. Anecdotal evidence suggests successful medical management of an enteric fistula due to CMV without the need for surgery. 
